# Highly variable hearing loss due to *POU4F3* (c.37del) is revealed by longitudinal, frequency specific analyses

**DOI:** 10.1038/s41431-023-01358-0

**Published:** 2023-04-19

**Authors:** Sushma Singh, Cindy Penney, Anne Griffin, Geoffrey Woodland, Salem Werdyani, Tammy A. Benteau, Nelly Abdelfatah, Jessica Squires, Beverly King, Jim Houston, Matthew J. Dyer, Nicole M. Roslin, Daniel Vincent, Pascale Marquis, Darren D. O’Rielly, Kathy Hodgkinson, Taylor Burt, Ashley Baker, Susan G. Stanton, Terry-Lynn Young

**Affiliations:** 1 Communication Sciences and Disorders and National Centre for Audiology, Western University, Elborn College, 1201 Western Road, London, ON Canada; 2 Centre for Translational Genomics, Health Sciences Centre, 300 Prince Philip Drive, St. John’s, NL Canada; 3 Faculty of Medicine, Health Sciences Centre, Memorial University, 300 Prince Philip Drive, St. John’s, NL Canada; 4 Central Health, Gander, NL Canada; 5 The Centre for Applied Genomics, The Hospital for Sick Children, Peter Gilgan Centre for Research and Learning, 686 Bay Street, Toronto, ON Canada; 6 Canadian Centre for Computational Genomics, McGill University, 740 Dr. Penfield Avenue, Montréal, QC Canada

**Keywords:** Medical genetics, Genetics research, Neurological disorders

## Abstract

Genotype-phenotype correlations add value to the management of families with hereditary hearing loss (HL), where age-related typical audiograms (ARTAs) are generated from cross-sectional regression equations and used to predict the audiogram phenotype across the lifespan. A seven-generation kindred with autosomal dominant sensorineural HL (ADSNHL) was recruited and a novel pathogenic variant in *POU4F3* (c.37del) was identified by combining linkage analysis with whole exome sequencing (WES). *POU4F3* is noted for large intrafamilial variation including the age of onset of HL, audiogram configuration and presence of vestibular impairment. Sequential audiograms and longitudinal analyses reveal highly variable audiogram features among *POU4F3* (c.37del) carriers, limiting the utility of ARTAs for clinical prognosis and management of HL. Furthermore, a comparison of ARTAs against three previously published families (1 Israeli Jewish, 2 Dutch) reveals significant interfamilial differences, with earlier onset and slower deterioration. This is the first published report of a North American family with ADSNHL due to *POU4F3*, the first report of the pathogenic c.37del variant, and the first study to conduct longitudinal analysis, extending the phenotypic spectrum of *DFNA15*.

## Introduction

Hearing loss (HL) is the most common sensory deficit globally. In countries with equitable access to comprehensive health care, Mendelian forms of HL account for most cases. So far, 154 deafness (DFN) genes have been identified to cause monogenic, isolated (non-syndromic) HL. In general, autosomal dominant (DFNA) genes cause postlingual and progressive sensorineural HL (SNHL) whereas autosomal recessive (DFNB) genes cause prelingual and severe-profound HL [1]. When a genetic diagnosis is made, genotype-phenotype correlations linking natural history to specific genetic etiology are valuable for prognosis and clinical management. Applications include family counseling, candidacy for cochlear implants [2] and emerging molecular therapies for HL [1]. Age-related typical audiograms (ARTAs) derived from cross-sectional analysis are useful to generalize genotype-phenotype correlations when the phenotypes are relatively homogeneous. However, phenotypic variability is both a hallmark of autosomal dominant sensorineural hearing loss (ADSNHL) and a clinical challenge as basic features (age of onset, audiometric configuration, rate of progression) can vary widely. For example, prognostic advice regarding “the family loss” based on the phenotypic history of other family members can be misleading. Depending on the genetic etiology, phenotypic variability is often seen not only with different variants in the same gene, but with the same variant, even between first degree relatives.

*POU4F3* (MIM*602460; NM_002700.3) encodes a hair cell specific member of the POU family transcription factor. Recent studies suggest that *POU4F3*, a direct transcriptional target of *ATOH1*, acts as a pioneer factor, binding to enhancers to make them epigenetically accessible and unlocking gene regulatory networks in the terminal differentiation of hair cells [3]. *POU4F3 (DFNA15)* was one of the first deafness genes to be identified in 1998, in an Israeli Jewish kindred where 12 members presented between 18–30 years of age with progressive ADSNHL [4–6]. So far, 33 *POU4F3* pathogenic variants have been reported along with large intrafamilial variation of HL, including the age of onset, audiogram configuration and more recently, presence of vestibular impairment.

Herein we report the first North American family co-segregating a pathogenic *POU4F3* variant, in this case a novel frameshift variant presenting a variable form of ADSNHL. Using audiometric data spanning eight decades, we comprehensively describe the natural history of *POU4F3* c.37del and compare it to published families with pathogenic *POU4F3* variants, uncovering new insights into interindividual variability and the limited utility of cross-sectional analysis and ARTAs when dealing with variable HL.

## Methods

### Family recruitment

**Fig. 1:**
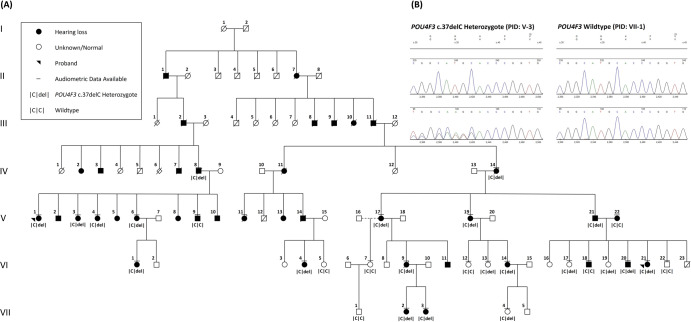
Pedigree of a North American kindred, initially recruited as two independent families (probands PID V-1, VI-21) showing co-segregation of a novel pathogenic *POU4F3* (c.37del) variant and autosomal dominant sensorineural hearing loss. **A** Seven-generation pedigree with 38 direct descendants of the pedigree founders with hearing loss are marked with a black symbol. **B** Electropherogram of the novel *POU4F3* c.37del frame-shifting variant (left panel, bottom) compared to reference sequence (left panel, top) and to an unaffected relative (right panel, bottom) compared to reference sequence (right panel, top). PID person ID in the pedigree.

As part of ongoing recruitment efforts in the Canadian province of Newfoundland and Labrador (NL), a seven-generation ADSNHL kindred was identified by genealogically connecting two independently recruited families (probands PID V-1 and VI-21, Fig. 1A). Informed consent was obtained to access medical and audiometric records and collect biological samples (protocol #01.186, Human Research Ethics Board, St John’s, NL, Canada).

### Audiometric classifications

The type and severity of HL was classified according to the GENDEAF criteria [7–9]. Calculation of pure tone average based on air-conduction thresholds at the four speech frequencies (PTA4 at 0.5, 1, 2, 4 kHz) was used to classify HL severity where PTA4 of <20 dB was classified as within normal range, 20–40 dB as mild HL, 41–70 dB as moderate HL, 71–95 dB as severe and >95 dB as profound HL [7]. Classification of SNHL required a bone-conduction threshold mean of *≥*20 dB and air-bone gap mean of <15 dB (averaged over 0.5, 1 and 2 kHz) [7, 8]. Subjects with PTA4 ≥ 20 dB were considered affected. A questionnaire adapted from the Harvard Centre for Hereditary Hearing Loss, which includes vestibular screening, was administered to multiple affected family members.

### Targeted sequencing, genome-wide genotyping and linkage analysis

Genomic DNA was isolated from peripheral blood [10] or saliva samples (Oragene kit, DNA Genotek Inc.). We used Sanger sequencing to screen probands for DFNA/B variants previously identified in the NL population (data not shown). A genome-wide SNP assay was done on selected members of the first recruited branch of the extended kindred (8 affected, 1 unaffected) using the Human610-Quad chip (Illumina Inc., San Diego, California, USA). Parametric linkage analysis was tested under an AD model with an estimated disease allele frequency of 0.0025 and penetrance of 99% (Merlin 1.1.2).

### Whole exome sequencing (WES) and segregation analysis

We next selected 14 subjects (7 affected, 1 unaffected, 6 unknown status) for WES. Exome library preparation was made with the Nextera DNA kit (Illumina Inc.) according to the manufacturer’s protocol. Libraries were multiplexed and sequenced using a S2 Reagent Kit (200 cycles) on the NovaSeq 6000 (Illumina Inc.). For both annotation and variant filtering, we used an in-house pipeline and multiple online databases (gnomAD, ESP6500, 1000 Genomes, ExAC) and prediction programs (SIFT, PolyPhen-2, Mutation Taster, PANTHER). Variants residing in *DFNA* genes with a maximum prediction score were validated by Sanger sequencing (plus intron/exon boundaries). PCR products were purified with ExoSAP-IT (Thermo Fisher Scientific, Waltham, Massachusetts, USA) and prepared for sequencing using BigDye XTerminator^TM^ Purification Kit (Thermo Fisher Scientific). Sequence data was analyzed by Mutation Surveyor (ver 5.12, SoftGenetics, State College, Pennsylvania, USA) and Alamut Visual (ver 2.11, Interactive Biosoftware, Saint Sulpice, Switzerland). Variants that co-segregated with SNHL in both branches of the extended kindred were screened in 187 HL probands and 71 ethnically-matched controls from NL, Canada.

### Cross-sectional and longitudinal linear regression

Binaural pure tone air conduction octave thresholds (most recent or only audiogram) were used to calculate frequency-specific linear cross-sectional regression analyses (thresholds on age). We calculated the annual threshold deterioration (ATD), representing the loss in dB/year and the coefficient of determination (*R*^2^) representing inter- and intraindividual variability at each octave frequency (Analysis Tool Pack, Microsoft Excel v16.0). We then compared the cross-sectional regression analysis of the North American kindred with three families (1 Israeli Jewish, 2 Dutch) [5, 9, 11].

To determine the rate of HL progression over time for individual members of the North American kindred, we did frequency and ear specific longitudinal linear regression analysis of pure tone thresholds for those with three or more sequential audiograms spanning 3 plus years. We used Analysis Tool Pack (Microsoft Excel v16.0) for all statistical analyses.

### Age-related typical audiograms (ARTAs)

For the North American kindred, we used frequency specific cross-sectional regression analysis to derive ARTAs at 10 year intervals spanning 1st–8th decades [12]. We derived two separate ARTAs, the first ARTA included all c.37del carriers irrespective of affection status (combined ARTA), and the second excluded the c.37del unaffected (non-penetrant) carriers. We then compared the combined ARTAs of the North American kindred (c.37del) with the ARTAs of the original Israeli Jewish family (884del8) [4–6] and two Dutch families (L829F and L223P) [9] previously evaluated by de Heer et al. [11].

## Results

### ADSNHL is caused by a novel variant in *POU4F3* (*DFNA15*)

Targeted Sanger sequencing for known *DFNA/B* pathogenic variants in the NL population yielded no hits. Genome-wide genotyping and linkage analysis on one branch of the North American kindred yielded three equally positive “logarithm of the odds” scores (LOD = 2.08) suggestive of linkage on chromosomes 2, 5 and 6. Subsequent WES identified 59 variants (40 SNPs, 19 indels, data not shown). Of these, only a novel variant, *POU4F3* NM_002700.3:c.37del, resided in a known *DFNA* gene. *DFNA15* (*POU4F3*) is located within one of the three linked genomic intervals (chr. 5q32). The *POU4F3* c.37del variant is predicted to create the frameshift p.(His13Thrfs*71) and appears to be novel, as it has not been reported in ClinVar, ClinGen, or gnomAD. Cascade sequencing revealed vertical transmission of the heterozygous *POU4F3* c.37del variant and co-segregation with HL on both branches of the extended kindred (Fig. 1). Targeted sequencing of 187 HL probands and 71 ethnically-matched population controls yielded no further positive hits. Based on the experimental evidence, *POU4F3* c.37del is classified as pathogenic, meeting both conservative (PVS1, PM2, PP1_moderate, PP3) and less conservative (PVS1, PM2, PP1_strong, PP3) criteria according to the American College of Medical Genetics and Genomics (ACMG) [13, 14].

### POU4F3 c.37del causes a variable ADSNHL with no apparent vestibular involvement

Cascade sequencing identified 21 family members heterozygous for *POU4F3* c.37del. Four carriers (PIDs IV-8, V-17, V-21, VI-19) were subsequently excluded from the regression analyses for various reasons (Table 1) which yielded 147 pure tone audiograms available for further analyses. Of the 17 *POU4F3* c.37del carriers, 14 carriers (PIDs IV-14, V-1, V-3, V-4, V-6, V-19, VI-1, VI-4, VI-9, VI-14, VI-20, VI-21, VII-2, VII-3) were classified as affected and 3 (PIDs VI-13, VI- 17, VII-4) as unaffected (non-penetrant) (Table 1 and Fig. 1). Audiograms for both North American (NA) probands (PIDs V-1 and VI-21) and one unaffected *POU4F3* c.37del carrier (PID V1-13) are provided (Supplementary Fig. 1A). The severity of HL is summarized using the final (most recent) audiogram (Table 1) and for *POU4F3* c.37del carriers with multiple audiograms, first audiograms are included. The three unaffected carriers were 5, 30, and 33 years of age at their final audiometric assessment. Although PID V1-13 reports good hearing and is unaffected in the 4th decade according to GENDEAF PTA4 criteria, a slight bilateral HL at 8 kHz is apparent on the final audiogram, with thresholds at 30 dBHL and 25 dBHL for the right and left ears respectively (Supplementary Fig. 1A). Responses to the questionnaire and/or case interview indicate that vestibular disturbances are not a central component of the phenotypic spectrum of *POU4F3* c.37del.

**Table 1:** Audiogram summary for each family member heterozygous for the POU4F3 (c.37delC) mutation.

	Final audiogram (better ear)				First audiogram (better ear)				Color code in Fig. 2
PID	Age (years)	Ear	4FPTA	Classification	AGE (years)	Ear	4FPTA	Classification	
VII-4	5^a^	L	12.5	Normal	–	–	–	–	Dash gray
VI-17	30^a^	R^b^	10	Normal	–	–	–	–	Dash light green
VI-13	33	R^b^	17.5	Normal	10	L	6.25	Normal	Solid orange
VI- 20	23^a^	R	25	Mild	–	–	–	–	Dash orange
VII-2	24	R	38.75	Mild	3	R	3.75	Normal	Solid purple
VI-21^g^	22	L	48.75	Moderate	5	L	20	Mild	Solid gray
VII- 3	26	R	51.25	Moderate	3	R	22.5	Mild	Solid green
VI-14	37	L	60	Moderate	19	L	37.5	Mild	Solid pink
VI-4	39	R	67.5	Moderate	29	R	46.25	Moderate	Solid blue
V-19	55	R	55	Moderate	44	R	58.75	Moderate	Dash blue
V-1^h^	60	L	56.25	Moderate	28	R	8.75	Normal	Solid dark red
V-4	75^a^	R	93.75	Severe	–	–	–	–	Solid olive green
VI-1	42	R^b^	92.5	Severe	30	R	40	Mild	Solid black
V-6	49^a^	R	86.25	Severe	–	–	–	–	Solid gold
V-3	56^a^	R^b^	87.5	Severe	–	–	–	–	Dash purple
IV-14	79	L	80	Severe	72	L	72.5	Severe	Solid light green
VI-9	39	L	97.5	Profound	8^f^	R	35	Mild	Solid red
IV-8^c^	75	–	–	–	–	–	–	–	
V-17^d^	50	–	–	–	–	–	–	–	
V-21^e^	–	–	–	–	–	–	–	–	
VI-19^e^	–	–	–	–	–	–	–	–	

Hearing loss severity classification based on GENDEAF criteria using 4FPTA (averaged dBHL thresholds at 0.5, 1, 2 and 4 kHz) of the better ear on final audiograms (Mazzoli et al. [7]).

*R* right ear, *L* left ear.

^a^Only one audiogram.

^b^Same 4FPTA for R and L, R ear data utilized for analysis.

^c^Audiometric data excluded (prominent history of noise exposure not shared by any other family members).

^d^Audiometric data excluded (thresholds are identified as tactile rather than auditory responses to sound).

^e^No audiometric data.

^f^The first audiogram showed mixed hearing loss with flat tympanometry.

^g^Proband first family.

^h^Proband second (related) family.

### Age of onset of hearing loss is variable in POU4F3 c.37del carriers

**Fig. 2:**
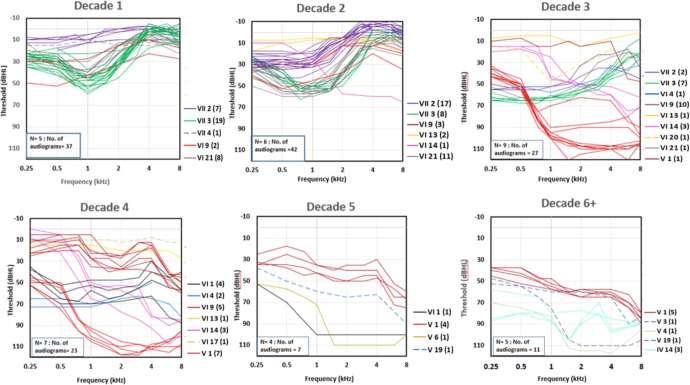
Decade-wise audiograms for *POU4F3 c.37delC* carriers in the North American kindred show variability in progression and severity. Each *POU4F3 c.37delC* carrier is represented by a unique colored line to track hearing loss progression. The unique PID (plus number of audiograms) is provided to the right of each graphic margin. The text box (bottom left) in each graphic gives the number (*N*) of *POU4F3 c.37delC* carriers and the total number of audiograms that were available per decade. Only a subset of *POU4F3 c.37delC* carriers have audiograms for each decade.

Initial audiograms for *POU4F3* c.37del carriers were obtained at various stages in the clinical course and range from normal hearing to profound hearing loss. For those with early onset hearing loss, the precise age of onset cannot be determined. However, the initial audiograms of PIDs VII-3, VI-21 and VI-9 confirm hearing loss during the first decade (Table 1 and Fig. 2). PID VII-3 exhibits hearing loss during infancy based on hearing thresholds obtained at 3 years of age (Table 1 and Fig. 2), with the possibility of prelingual onset. The first audiograms acquired for PIDs VI-21 and VI-9 confirm the presence of hearing loss during childhood, at 5 and 8 years of age respectively. In contrast, several normal hearing tests during the first decade confirm a delayed onset of hearing loss until 11 years of age for PID VII-2 (Fig. 2).

Adult onset of hearing impairment was established for PID V-1, who presented with hearing in the normal range according to the initial audiogram PTA4, obtained late in the third decade. Nevertheless, this initial audiogram revealed an early indication of auditory damage given the mild loss at 8 kHz, which progressed to affect hearing sensitivity across the frequency range in subsequent decades (Supplementary Fig. 1A). For most family members inheriting one copy of *POU4F3* c.37del, the exact age of hearing loss onset could not be confirmed audiometrically, given that hearing loss was present on the first audiogram acquired during adulthood.

### Variability in the configuration and course of deterioration across frequencies in POU4F3 c.37del carriers

**Fig. 3:**
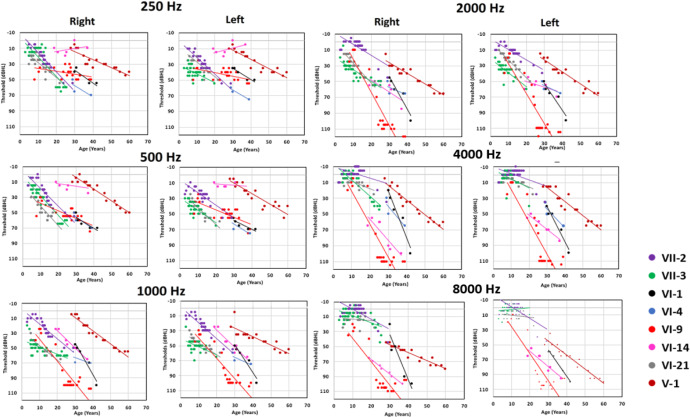
Longitudinal linear regression analyses of pure tone hearing thresholds for eight affected *POU4F3* c.37del carriers. Each panel provides serial pure tone air conduction thresholds as a function of age, and longitudinal linear regression results at each octave frequency from 0.25–8 kHz; right and left ear results are shown separately.

Sequential audiograms were available for eight affected *POU4F3* c.37del carriers (V-1, VI-1, VI-4, VI-9, VI-14, VI-21, VII-2, VII-3). Pure tone air conduction thresholds were analyzed at each frequency, separately for the right and left ears. Sequential audiograms and individual longitudinal regression analyses with corresponding annual threshold deterioration (ATD) reveal striking variability in the age and audiometric configuration, and in the subsequent course of hearing deterioration across the frequency range (Fig. 3 and Supplementary Table 1).

The early audiograms of family members with childhood hearing loss reveal distinct audiometric configurations across the frequency range. All four P*OU4F3* c.37del carriers with hearing loss identified during their 1st decade exhibit an unusual audiogram configuration involving the low and/or mid-frequencies (proband VI-21 and PIDs VI-9, VII-2, VII-3) with changes in audiogram configuration over the clinical course (Fig. 2).

For example, proband VI-21 at 5 years of age had a mild low-frequency loss exclusively, which then progressed to include the mid-frequencies while sparing higher frequencies at 22 years (Supplementary Fig. 1A). However, PID VII-3 presented with a mid-frequency notch (750–1000 Hz) configuration, which first progressed to a low-frequency configuration, and then later declined in the high frequencies (Fig. 2 and Supplementary Fig. 1B). Longitudinal regression analyses confirm this interindividual variability in threshold decline across the frequency range. PIDs VI-21 and VII-2 show high ATDs across the low and mid-frequency range, while PID VII-3 shows high ATDs at low frequencies only (Fig. 3 and Supplementary Table 1). On the other hand, PID VI-9 experienced rapid decline across the mid-upper frequency range, transforming their initial low-mid-frequency profile into a sloping toward high frequency configuration (Fig. 2 and Supplementary Fig. 1B) and longitudinal regression of the sequential audiograms shows this higher rate of deterioration in the mid and high frequencies (ATDs >4 dB/year at 2 and 4 kHz) (Supplementary Table 1).

In contrast, two P*OU4F3* c.37del carriers with later hearing loss diagnoses (proband V-1 and PID VI-1) show the opposite configuration, with an initial high frequency hearing loss (Fig. 2 and Supplementary Fig. 1). Proband V-1 at 28 years of age had reduced hearing sensitivity only at 8 kHz and was classified as normal hearing (Supplementary Fig. 1A). Hearing thresholds subsequently progressed to a moderate flat configuration by 60 years following a slow rate of progression across all frequencies (ATD 1–1.8 dB/year) (Supplementary Table 1). On the other hand, PID VI-1 experienced a rapid progression (ATD > 4 dB/year) in the mid and high frequencies while PID VI-4 showed high deterioration at 4 kHz only (ATD 2.8 dB/year) (Fig. 3 and Supplementary Table 1). PID VI-14 was not identified until the 2nd decade with a high frequency hearing loss; low-frequency hearing sensitivity was preserved into the late 4th decade, resulting in a steeply sloping audiogram configuration (Fig. 2 and Supplementary Fig. 1B).

Longitudinal threshold data also revealed deterioration rates that fluctuated with age and frequency (Fig. 3). Two affected *POU4F3* c.37del carriers (PIDs VI-9, VII-3) experienced gradual progression alternating with periods of rapid deterioration (Fig. 2 and Supplementary Table 1). A rapid loss of mid to high frequencies was experienced by PID VI-9 between 19 and 23 years of age, and another between 23 and 27 years of age in the mid-frequencies from 1000 to 4000 Hz (Supplementary Fig. 1B). The hearing loss progression in PID VII-3 also showed two periods of rapid change, the first between 14 and 20 years of age at low frequencies (250–500 Hz) and then between 20 and 26 years of age at high frequencies (>4000 Hz) (Supplementary Fig. 1B). Although PID VI-14 experienced gradual progression across most of the frequency range (ATD ~ 2 dB/year), fluctuating hearing sensitivity was apparent at 0.25 kHz in the late 3rd decade, with an ATD of −0.3 dB/year at this frequency (Fig. 3 and Supplementary Table 1).

Unfortunately, the audiometric configuration and threshold progression could not be determined for PIDs V-3, V-4, V-6, IV-14; their initial audiograms, obtained between the 5th–8th decades, show a flat hearing profile with sensitivity approaching the audiometric ceiling indicating substantial auditory damage (Fig. 2).

### Cross-sectional linear regression analyses of final visit audiograms: ATD and ARTA

In order to compare and contrast phenotypic data between the NA kindred and the Israeli Jewish family (884del8) and two Dutch families (L829F and L223P), we used only final visit audiograms in cross-sectional linear regression analyses and corresponding ATDs and ARTAs.

For the NA kindred, cross-sectional analyses produce frequency specific threshold deterioration rates ATDs ranging from 0.36–1.32 dB/year. However, lower *R*^2^ values from 0.25–1 kHz imply high interindividual variability in threshold deterioration rates in this frequency range (Supplementary Fig. 1C and Supplementary Table 2).

**Fig. 4:**
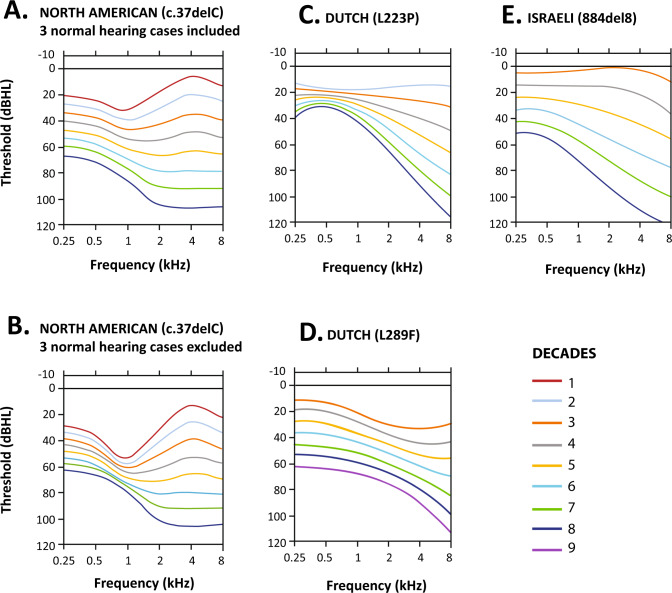
Comparison of age-related typical audiograms (ARTAs) across four *DFNA15/POU4F3* pedigrees. **A** ARTA for the North American kindred range from the 1st to 8th decade including all *POU4F3* c.37del carriers (17 clinically affected including 3 with normal hearing). **B** ARTA for the 14 clinically affected c.37del carriers. **C** ARTA from a Dutch family with the L223P variant. **D** ARTA for a Dutch family with the L289F variant. **E** Israeli Jewish family with the 884del8 variant. All analyses are based on original threshold data without correction for presbycusis. ARTAs adapted from this figure (de Heer et al. [11]) with permission.

ARTAs generated from these regression equations indicate that *POU4F3* (c.37del) carriers experience an early onset phenotype beginning in the 1st decade (Fig. 4A) with an unusual mid-frequency notch initially, followed by gradual decline and flattening of the audiogram, and then a more rapid high frequency progression later in life. The same phenotype pattern was obtained, whether or not the 3 unaffected *POU4F3* c.37del carriers were included, albeit with differences in the projected ages of HL onset and progression (Fig. 4A, B). Although the same statistical methods [12] were used, ARTAs previously generated by de Heer et al. [11] demonstrate distinctly different phenotypes, with adult-onset hearing loss delayed until the 3rd decade (2 Dutch families with L829F and L223P variants) or 4th decade (Israeli family with 884del8 variant) and gently sloping or flat audiogram configurations initially (Fig. 4C–E).

We conclude that using ARTAs for predicting the clinical course of hearing loss due to *POU4F3* is of limited utility because it obscures the highly variable phenotype. Comparison of ARTAs across published studies ostensibly show distinct genotype-phenotype correlations for these four pathogenic variants in the *POU4F3* gene. These interfamilial variations are influenced by differences in the number of cases, age distribution and HL severity of cases used to create each family ARTA. However, given the high degree of interindividual variability, as confirmed by longitudinal analysis of the NA family, it is clear that ARTAs have limited utility in the clinical management of *POU4F3* cases.

## Discussion

We report the first North American kindred with ADSNHL due to *POU4F3* (*DFNA15*) and identify a novel pathogenic variant (*POU4F3* c.37del), the result of a comprehensive molecular approach including targeted sequencing, linkage analysis and WES. Working closely with affected families with an emphasis on mutual benefit, complete clinical ascertainment of both affected and unaffected family members, and the use of genealogy to connect independently recruited families were also essential ingredients to a successful outcome. *POU4F3* is noted for remarkably large variation of the hearing loss phenotype, including the age of onset and audiogram configuration with flat, mid and high frequency hearing losses across families and within the same family [4, 15–30]. The presence of vestibular impairment has also been observed in some families [9, 18, 19]. Longitudinal data on the North American kindred was used to fully describe the natural history and compare it with previously published families.

ARTAs derived from cross-sectional analysis are often used to predict HL progression in cases with the same underlying genetic etiology [31, 32]. However, in this NA kindred, ARTAs predicting early mid-frequency hearing impairment do not reflect the natural course for *POU4F3* c.37del carriers experiencing later onset and other audiogram configurations. Longitudinal audiogram analyses confirm that HL onset and frequency-specific configuration and progression due to *POU4F3* c.37del is highly variable between carriers. Some carriers exhibit a low-frequency configuration or mid-frequency notch in the audiogram early in the clinical course, both of which are rare phenotype presentations regardless of whether the hearing loss is an acquired or genetic etiology [31, 32].

De Heer et al. [11] were the first to investigate the onset and course of *DFNA15* HL for different genotypes by generating ARTAs from Dutch (L829F and L223P) and Israeli (884del8) cross-sectional regression equations. However, the age distribution of hearing data skewed toward late middle age and older introduced bias, with little or no representation of the early-stage disease process [6, 9, 11]. The authors also describe phenotypic variability based on documented audiograms that deviate from their corresponding ARTA profiles in each study. Both low and mid-frequency audiogram configurations were reported for *POU4F3* carriers in the Dutch families and are similar to auditory phenotypes expressed by several members of the NA kindred.

Our findings confirm that variable expressivity is the defining feature of DFNA15 (*POU4F3*), and that heterozygous individuals (carriers) may exhibit low or mid-frequency audiogram configurations in early clinical stages. It is particularly noteworthy that these rare configurations are not reliably detected by physiological newborn hearing screening and may also go undetected in early childhood. Furthermore, ARTA profiles based on cross-sectional analyses are unreliable for clinical prognosis and management of *POU4F3* patients. Understanding the range of variability of hearing loss associated with *POU4F3* can facilitate early diagnosis and intervention, while also supporting appropriate clinical management strategies. Combined with genetic identification of at-risk individuals, knowledge of phenotypic variability is a powerful tool for patient-centered HL management.

How the novel mutation identified in this study leads to SNHL is not known but recent studies define a pivotal role for POU4F3 in both sensory hair cell development and maintenance. Auditory hair cells are sensitive to aging, noise exposure, infection and ototoxic drugs and SNHL caused by damaged hair cells is permanent. The mammalian auditory sensory epithelium (the organ of Corti) is a cellular mosaic of sensory hair cells and non-sensory supporting cells. After a damaging event, hair cells can spontaneously regenerate from cochlear supporting cells, but only within the first week of life. The ATOH1 gene has been intensely studied as the “transcriptional master regulator” of differentiation of hair cells as a potential therapeutic strategy for SNHL [33]. During development, hair cells and supporting cells arise from postmitotic cells in a temporal basal-to-apical gradient. POU4F3, a member of a newly recognized class of the so-called pioneer transcription factors, binds to DNA that is silenced within nucleosomes [34]. POU4F3 is the only pioneer transcription factor in the cochlea and orchestrates a hierarchy of gene expression through its initial interaction with ATOH1. POU4F3 acts in a feed-forward mechanism in which ATOH1 first stimulates expression of POU4F3, which subsequently acts as a pioneer factor to provide access to closed ATOH1 enhancers [3]. When ATOH1 is initially up regulated, most of its expression enhancer sites exist within closed chromatin. POU4F3 binds to regions of DNA located near ATOH1 enhancer targets within the closed chromatin, beginning the process of opening up the chromatin to additional transcription factors. ATOH1 expression in the developing mouse cochlea can also induce hair cell regeneration, however this ability reduces as the cochlea matures [35]. Cisplatin, a well-known chemotherapy drug with limited use due to high ototoxicity is more damaging in the presence of gene mutation in POU4F3, suggesting POU4F3 protects hair cells from inflammatory programmed cell death (pyroptosis) in mice, activated through the NLRP3/Caspase-3/GSDME pathway [36] and that these surviving hair cells are capable of self-repair [37, 38]. The highly variable age of onset of SNHL due to *POU4F3* (c.37del) is consistence with a dysregulation of hair cell maintenance in combination with modifying effects of both environmental exposures and genetic background.

Strengths of this study include comprehensive molecular analysis to identify the genetic etiology, near complete clinical ascertainment of both affected and unaffected family members, genealogical work to connect pedigrees, retrieval of longitudinal data and the use of various phenotypic analyses to reveal the extent of intrafamilial variability. Limitations include the inability to calculate the penetrance of *POU4F3* c.37del without population-based data. Penetrance does appear to be high in the NA kindred, which may not be representative in all families due to ascertainment bias, where the most dramatically affected families are the ones that garner attention from geneticists. As is characteristic of AD conditions and specifically HL due to *POU4F3*, penetrance is age-related. Of the three non-penetrant *POU4F3* carriers classified as normal hearing (according to GENDEAF PTA4 criteria), two carriers are in their 30s (one with mild threshold loss at 8 kHz bilaterally) and the other is a 5-year-old with normal hearing sensitivity. Because penetrance is age-related, it is possible that unaffected *POU4F3* c.37del carriers will be affected later. Given the variability in phenotype, including both inter- and intraindividual variation that is characteristic of *POU4F3* as revealed in this and other studies [4, 15–28, 30], and the broad range of onset from 1–59 years recently reported in a Japanese study [18], it is critical that non-penetrant North American *POU4F3* c.37del carriers be closely monitored.

Large extended kindreds with HL co-segregating with the same pathogenic variant cascading through the generations provide rich inputs for genotype-phenotype correlations that are valuable for accurate prognosis and better clinical management. This is the first detailed analysis of the natural history of *POU4F3* in multiple subjects from the same family with longitudinal data. Interfamilial, interindividual, and intraindividual variability is a prominent feature of *DFNA15*/*POU4F3* phenotype which limits the prognostic value of ARTAs based on final visit audiograms and cross-sectional data. Future studies include using advanced phenotypic measurements such as otoacoustic emissions (OAEs) and auditory evoked potentials (AEPs) to identify the site of lesion in the auditory system during early stages of the disease. To improve clinical management and counselling of families, it will be important via population-based studies to calculate age-specific penetrance (per decade) for pathogenic variants in *POU4F3* and other *DFNA* genes to address inherent ascertainment bias in gene discovery studies like this one.

## Supplementary information

Suppl. Fig. 1

Suppl. Table 1

Suppl. Table 2

## Data Availability

The datasets generated and analyzed during the current study are available from the corresponding author on reasonable request and have been uploaded to ClinVar (accession SCV002556358).
