# Supplementary figures and images for: Highly variable hearing loss due to POU4F3 (c.37del) is revealed by longitudinal, frequency specific analyses
Source: Eur J Hum Genet. 2023 Apr 19;31(7):815–23. doi: 10.1038/s41431-023-01358-0 (PMC10325993; doi:10.1038/s41431-023-01358-0)

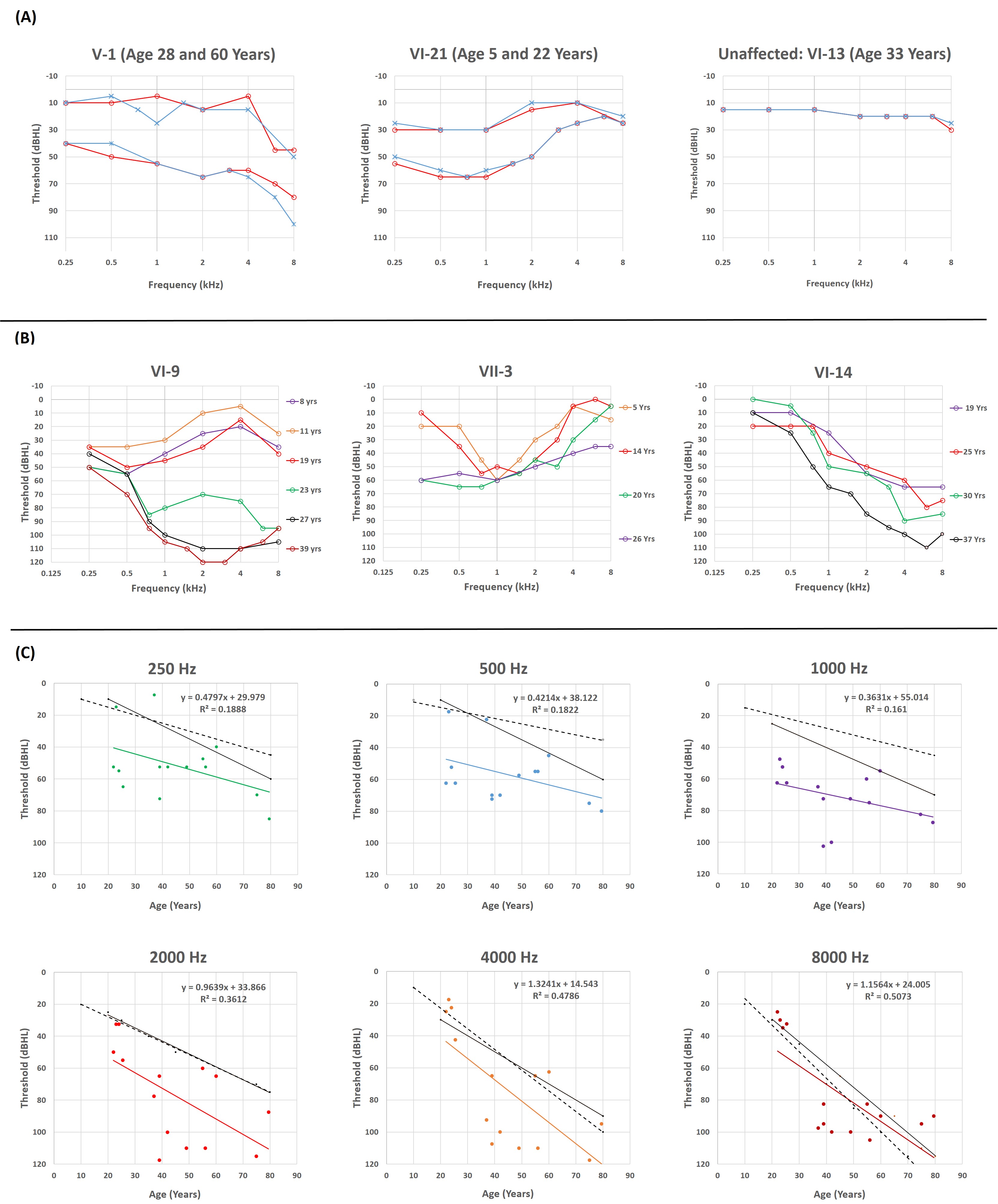

Supplement: Supplementary file 1 — Suppl. Fig. 1 [file 41431_2023_1358_MOESM1_ESM.jpg]
